# Challenges and Approaches to Green Social Prescribing During and in the Aftermath of COVID-19: A Qualitative Study

**DOI:** 10.3389/fpsyg.2022.861107

**Published:** 2022-05-16

**Authors:** Alison Fixsen, Simon Barrett

**Affiliations:** ^1^Department of Psychology, School of Social Sciences and Humanities, University of Westminster, London, United Kingdom; ^2^Population Health Sciences Institute, Newcastle University, Newcastle upon Tyne, United Kingdom

**Keywords:** social prescribing, green prescribing, COVID-19, mental health outcomes, health inequalities

## Abstract

The last decade has seen a surge of interest and investment in green social prescribing, however, both healthcare and social enterprise has been impacted by the COVID-19 crisis, along with restricted access to public green spaces. This study examines the challenges and opportunities of delivering green social prescribing during and in the aftermath of COVID-19, in the light of goals of green social prescribing to improve mental health outcomes and reduce health inequalities. Thirty-five one-to-one interviews were conducted between March 2020 and January 2022. Interviewees included Link Workers and other social prescribers, general practitioners (GPs), managers, researchers, and volunteers working in urban and rural Scotland and North East England. Interview transcripts were analyzed in stages, with an inductive approach to coding supported by NVivo. Findings revealed a complex social prescribing landscape, with schemes funded, structured, and delivered diversely. Stakeholders were in general agreement about the benefits of nature-based interventions, and GPs and volunteers pointed out numerous benefits to participating in schemes such as parkrun. Link Workers were more circumspect about suggesting outdoor activities, pointing out both psychological and practical obstacles, including health anxieties, mobility issues, and transport deficits. Exacerbated by the pandemic, there was a way to go before older and/multi-morbidity clients (their largest cohort) would feel comfortable and safe to socialize in open air spaces. Our findings support the premise that time spent in open green spaces can alleviate some of the negative mental health effects compounded by the pandemic. However, the creation of healthy environments is complex with population health intrinsically related to socioeconomic conditions. Social disadvantage, chronic ill health and health crises all limit easy access to green and blue spaces, while those in the most socially economically deprived areas receive the lowest quality of healthcare. Such health inequities need to be borne in mind in the planning of schemes and claims around the potential of future nature-based interventions to reduce health inequalities.

## Introduction

Accelerated by the COVID-19 pandemic, the social and material world has undergone rapid change, including an increased focus on the relationship between health and the natural world. One way of encouraging people to improve their health and wellbeing “naturally” is through green social prescribing, an approach whereby a general practitioners (GPs) or another professional – often *via* an assigned intermediary known as a Link Worker – navigates a patient or client toward a nature-based intervention and activity such as a local walking and running program, community gardening or other outdoor project. A small body of literature has now focused on the work of nature-based social prescribing outside of pandemic conditions (Leavell et al., 2019; Robinson et al., 2020). Our study is one of the first to qualitatively examine the views and experiences of professional and volunteer social prescribing stakeholders with regard to green prescribing during the COVID-19 pandemic period and the future of social prescribing. Using data from 35 one-to-one interviews conducted from March 2020 to January 2022, this study critically examines the challenges and opportunities for delivering green social prescribing during the pandemic – and how they could be addressed in the aftermath of the COVID-19 pandemic – and considers them in the light of the recent aims set out by the United Kingdom government to improve mental health outcomes and reduce health inequalities (Public Health England, 2021).

## Background

### An Overview of Social Prescribing

The term “social prescribing” describes the practice of supporting people to access non-medical forms of health and support in the local community (Lejac, 2021). The stated goals of social prescribing are broad and include; reducing health inequalities (Mercer et al., 2019), strengthening primary-care/third-sector partnerships (Brandling and House, 2009) and lessening GP burden (Mercer et al., 2019). Within the overall framework of social prescribing, schemes follow different models; some receive funding from governments, others are funded and managed through private or charity sectors, while many are mixture of both. The most common referral model in the United Kingdom is the one where a Link Worker or designated Social Prescribing Advisor acts as a mediator between a referrer (typically a GP) and local service provider (Islam, 2020). Under schemes with the capacity, referrals by other professionals, such as social workers and job center employees, as well as self-referrals, are also possible (Mercer et al., 2019; Fixsen et al., 2020). While anyone with non-medical needs might benefit from social prescribing it is particularly targeted at people with long-term health conditions (Moffatt et al., 2017) and/or mental health issues (Pescheny et al., 2018; Gibson et al., 2021).

Variations in the terminology around social prescribing be confusing; the term “social prescribing” is sometimes used interchangeably with “community referral” and “linking scheme” (Hassan et al., 2020), while Link Workers may be known by an alternative title, such as “Social Prescribing Coordinator/Advisor” or “Community Navigator.” What counts as a “social prescription” is diverse but may include an exercise and physical activity program (e.g., a gardening project or walking or cycling group), or it may be another social activity such as painting, dance, music, or poetry. Many clients come with complex socioeconomic needs, hence coordinators may also refer people to support services concerned with mental health, employment, finances, and housing (Husk et al., 2016; Fixsen and Polley, 2020; Polley et al., 2020).

### Social Prescribing in the United Kingdom

In the United Kingdom, the National Health Service (NHS) is the umbrella term for the four health systems of England, Scotland, Wales, and Northern Ireland, however, since devolution there has been increasing divergence of these health systems (The Health Foundation, 2014). Scotland’s NHS is a separate body from the other public health systems in the United Kingdom, with primary and secondary care integrated in Scotland. All the United Kingdom governments subscribe to social prescribing and provide funding for projects and Link Workers, in England *via* Primary Care Networks (PCNs) and in Scotland *via* the Primary Care Improvement Fund (PCIF) for Community Link Workers (CLWs).

Within both Scotland and England, the development of community-led approaches has been gradual and uneven. The term “social prescribing” was officially adopted by the United Kingdom Department of Health in 2006 (Department of Health, 2006), however, unofficial forms of social prescribing existed decades earlier, largely planned and delivered by neighborhood community and voluntary groups in association with interested local GPs (Dayson, 2017). In England, the NHS, 2014 Five Year Forward Plan identified the challenges facing contemporary health services, particularly the need for mental and physical health support to be holistically integrated around the patient (Lejac, 2021). The expansion of social prescribing in England has been significant; under the NHS Model of Personalized Care, over 900,000 people could be referred into an NHS social prescribing scheme by 2023/24 (NHS England, 2022a).

In Scotland, a 2007 government-commissioned report by the Scottish Development Centre for Mental Health identified social prescribing’s potential to become fully integrated as a patient pathway for primary care practices. The Scottish Links Worker Programme was piloted in Glasgow in 2011 and was later extended to other cities. NHS Health Scotland’s 2016 guidance paper on Social Prescribing in Mental Health similarly identified the potential for social referrals to support statutory services in addressing the many social factors that contribute to poor mental health. This was followed by Public Health Scotland’s 2018 release of social prescribing resources for primary health services, including case studies of good practice and guidelines for implementation (Lejac, 2021). Despite large increases in local social prescribing services, the present distribution remains patchy in both England and Scotland, leading for calls for the upscaling of social prescribing across both countries (Lejac, 2021; Morris et al., 2022).

At the present time, the United Kingdom leads the way in social prescribing, however, other parts of the world are increasingly investing in this approach. Social prescribing programs now exist in several European countries, the United States, Canada, New Zealand, and Brazil (Younan et al., 2020). The National Social Prescribing Academy, in collaboration with the World Health Innovation Summit (WHIS), the World Health Organization (WHO), and United Nations Global Sustainability Index Institute (UNSGII), set up the Global Social Prescribing Alliance, to promote Social Prescribing internationally and support the implementation of the United Nation’s Sustainable Development Goals (Global Social Prescribing Alliance, 2022).

Arguments in favor of social prescribing as a valuable non-medical form of health care seem compelling (Islam, 2020), however, the systematic evidence concerning effectiveness of social prescribing on reducing GP visits and increasing patient health and wellbeing is not strong (Bickerdike et al., 2017; Gibson et al., 2021). Comparisons between social prescribing schemes are problematic as they adopt different models of healthcare (Fixsen et al., 2020) and are situated in areas of greater or lesser socioeconomic need (Costa et al., 2021). Critics of social prescribing point to a reliance on community goodwill and use of individual-level health interventions to address socially and economically embedded issues, which demand the implementation of government-level interventions (Bickerdike et al., 2017; Mackenzie et al., 2020; Gibson et al., 2021). Nevertheless, there is some evidence supporting the health and social benefits of social prescribing to individuals and groups in specific communities (Dayson and Bennett, 2018; NHS England, 2022b), while its role in extending the boundaries of traditional general practice and strengthening community-professional partnerships has been substantiated by various studies (Mercer et al., 2019; Tierney et al., 2020). The precise ending of the pandemic is impossible to predict, however, a report commissioned by the Royal Society of Edinburgh’s Post COVID-19 Futures Commission, explores the potential for social prescribing to contribute to the recovery of Scotland’s public service in the wake of the COVID-19 crisis. The report states that non-medical approaches could lessen the pressure on the NHS and other public services in the wake of COVID-19, but this is only if community partners are adequately resourced to deliver tailored support across Scotland (Lejac, 2021).

### Green Social Prescribing

One of the markers of the last decade has been a surge of interest in nature-based or “green prescribing,” whereby clients can access low or no-cost open-air activities, such as walking, running, gardening, or outdoor volunteering (Aerts et al., 2018; Leavell et al., 2019; Robinson et al., 2020). With data indicating that around 90% of health determinants derive from peoples’ lifetime social and physical environment rather than their health care provision (Carod-Artal, 2017) there is ample evidence to support this initiative. In 2013 the United Kingdom National Institute of Health and Care Excellence (NICE) recommended that primary care teams deliver tailored physical activity advice to inactive adults (NICE, 2013). July 2020, the United Kingdom government announced a £4,000,000 injection of funding to embed green social prescribing into local communities, expressly to help tackle the negative mental health impact of COVID-19 and reduce health inequalities (NHS England, 2022b).

Although establishing a direct causal relationship between green spaces and health has proved difficult, the vast majority of studies support the premise that green space has a beneficial effect on health and wellbeing (Lee and Maheswaran, 2011). Studies suggest that natural environments and green spaces provide ecosystems and services that are considered to enhance human health and well-being in multiple ways (Aerts et al., 2018). Increasing people’s exposure to, and use of, green spaces has been linked to increased social contacts and higher self-rated mental health, as well as reductions in physical health problems including heart disease, cancer and musculoskeletal conditions and obesity (Alcock et al., 2014; Nieuwenhuijsen et al., 2017; Fixsen and Polley, 2020). In one field experiment, gardening was found to promote neuroendocrine and affective restoration from stress more rapidly than other calming activities such as reading (Van Den Berg and Custers, 2011). There is emerging evidence that gardening may also be important in the prevention of falls by helping to maintain good gait and balance, and also in dementia prevention and cognitive decline (Buck, 2016).

Some of the earliest studies of green social prescribing have emerged from New Zealand, where this approach has been used for decades to encourage patients to be more active. Here, green social prescribing is implemented at the primary care level, with referrals shared between the practice and regional sports trusts (Hamlin et al., 2016). Findings from studies suggest good results, with patients taking part in green prescribing in New Zealand reporting greater perceived health benefits following the program and being more likely to meet current physical activity guidelines when compared to those not engaged in green prescribing (Sinclair and Hamlin, 2007; Hamlin et al., 2016).

One ‘‘green space’’ initiative that is now widely available in the United Kingdom and internationally is *parkrun*,^1^ a registered non-profit company that delivers free 5 km and other events for all ages, and junior parkrun events for 4–14 year olds, organized by volunteer teams and held in public spaces, including municipal parks and green environments (Wiltshire et al., 2018; Fleming et al., 2020; Hindley, 2020). Participants in parkrun can walk, run, jog, or volunteer as marshals or runner counters. Evidence concerning the benefits and attractiveness of parkrun for both runners and non-runners has been mounting, and schemes have increasingly linked up with local GP practices to improve the health and wellbeing of both patients and staff. Qualitative studies suggest parkrun to be “more than just a run in the park”; it is specifically designed to be inclusive, easily accessible and socially supportive (Hindley, 2020). As a socially situated “health practice,” as distinct from an individualized “health behavior” (Wiltshire et al., 2018), it aligns with the concept of social prescribing as a form of social capital (Polley et al., 2020). In one study, the least active participants from the most socioeconomically deprived areas reported the most improvement in activity and wellbeing levels (Quirk et al., 2021), suggesting the leveling up potential of parkrun. At the same time, social and health divisions can act as barriers to participating in outdoor activities like running, partly due to its association with certain cultural groups and body ideals. Fullagar (2016) identified fewer parkrunners from non-white British backgrounds, even in areas of high ethnic diversity. Schemes such as parkrun may inadvertently favor a young, more socioeconomically advantaged and a predominantly white client-base, even while taking measures to open their schemes up to everybody.

The United Kingdom government and NHS have advocated an expansion of green social prescribing to lessen pandemic effects on mental health and wellbeing (Public Health England, 2021). Multiple nature inspired initiatives have emerged to support people during and after the pandemic, including novel enterprises such as “green cafes” and nature inspired arts activities, designed to embed green social prescribing in the local community (National Academy for Social Prescribing, 2022). Recent funding from United Kingdom government departments and allied partners has resulted in a number of testing and learning sites projects for green social prescribing aimed at improving mental health outcomes, reducing health inequalities, reducing demands on the health and social care system and making such green projects more resilient and accessible (NHS England, 2022b). At the same time, studies suggest that constraints to green social prescribing exist, with fewer referrals or take up to green activities associated with higher levels of deprivation (Robinson et al., 2020). Added to this, the overall effects of COVID-19 on many communities in the United Kingdom has been both profound and unequal. Studies of previous pandemics confirm that the most disadvantaged people in society suffer most (Madden et al., 2020). In the light of the recent United Kingdom government aim to improve mental health outcomes and reduce health inequalities (Public Health England, 2021) in the current socioeconomic landscape, our article explores the opportunities for and barriers to green social prescribing during COVID-19 and as envisaged post-COVID-19, from the perspective of those involved in its delivery.

## Materials and Methods

A qualitative study design was used to explore barriers and enabling factors affecting the delivery of social prescribing services, including natured-based interventions, to those living in urban and rural areas of Scotland and the North of England during the pandemic.

Our aim was to elicit responses from a broad range of stakeholders involved in social prescribing delivery before and during COVID-19.

### Research Questions

The main questions guide our enquiry are:

• What are the challenges to and opportunities from delivering green social prescribing during and post-COVID-19?
• How do our findings align with plans to expand green social prescribing as a means of improving mental health outcomes and reducing health inequalities?

### Participants

In all, 28 stakeholders in social prescribing were interviewed between March 2020 and December 2021, 7 of them on 2 occasions, making a total of 35 interviews. Interviewees included 6 GPs, 11 Link Workers or their equivalent, along with managers, volunteers, and researchers. Several participants held more than one role (e.g., GP/third sector volunteer, researcher/third sector volunteer). All stakeholders had knowledge of, and/or associations with one or more of the following different schemes:

• Scotland – The Deep End CLW programme, managed by the Health and Social Care Alliance
• Scotland – SPRING social prescribing, funded by National Lottery Fund and managed by Scottish Communities for Health and Wellbeing (SCHW)
• Scotland – mPower Community Navigator project managed through the Special EU programmes body with match funding from Scottish Government
• England – The CLW programme, managed and funded through PCNs.

Table 1 gives the details of individual participants including dates of interviews, work role, and work locations.

**TABLE 1:** Key: table of participants with dates of interviews, role description, and work location.

Interview date	Role	Program
01/04/2020 18/02/2021	Manager	SPRING, Glasgow
05/04/2020	GP	Deep End GP practice, Glasgow
07/04/2020 02/02/2021	Community Link Worker	Deep End GP practice, Glasgow
09/04/2020	Community Link Worker	Deep End GP practice Glasgow
20/04/2020 12/02/2020	Social Prescribing Advisor	SPRING, Glasgow
21/04/2020 03/02/2021	GP	Deep End GP practice, Glasgow
28/04/2020 27/01/2021	Manager	CLW programme, Scotland
28/04/2020	Manager	SPRING, Scotland
28/04/2020	Researcher	Scottish Government
01/05/2020	GP	GP practice, Glasgow
05/05/2020	GP	Deep End GP practice, Glasgow
07/05/2020	Community volunteer	Third sector organization, Edinburgh
11/05/2020	GP	GP practice, Glasgow
14/05/2020 04/02/2021	Community Link Worker	Deep End GP practice, Glasgow
29/05/2020	Community volunteer	Third sector organization, Glasgow
30/05/2020	Social Prescribing Advisor	SPRING, Edinburgh
19/05/2020	Researcher	University, Scotland
24/05/2020	Manager	mPower, Scotland
26/05/2020	Manager	mPower, Scotland
04/06/2020	Community Navigator	mPower, rural Scotland
11/06/2020 05/02/2021	Community Navigator	mPower, rural Scotland
16/06/2020	Community Navigator	mPower, rural Scotland
18/06/2020	Researcher	University, Scotland
15/03/2021	Community Link Worker	Deep End GP practice, Glasgow
18/11/2021	Community volunteer	Third sector organization, NE England
14/12/2021	GP	GP practice, NE England
18/12/2021	Community Link Worker	GP practice, NE England
20/12/2021	Community Link Worker	GP practice, NE England

*GP, general practitioner.*

### Data Collection

A mixture of purposive and snowball sampling was used to recruit participants with different interests and involvements in social prescribing first in the Glasgow area, then rural Scotland and finally in the North of England. A loosely structured interview guide was designed for the interviews and adapted to suit the rapidly changing conditions at this time. Prospective participants were contacted by email by the first author, who explained the researchers’ backgrounds and interests in the study and attached a Participant Information and Consent Form. All those contacted agreed to be interviewed, although two interviews did not go ahead due to illness. Due to social distancing conditions, all interviews were conducted individually by the first author (an experienced qualitative researcher) *via* Skype, Teams, or telephone. Interviews were 30–60 min in length and were audio recorded.

### Data Analysis

Inductive thematic analysis was used to examine the initial set of data, and to amend and add to it based on data from follow up interviews. We used aspects of grounded theory, such as beginning the study without any recognized theoretical framework, using theoretical sampling in the selection of participants over time, breaking up data into words, phrases and sentences, and adopting a system of open and axial coding, searching for sensitizing concepts and making constant comparisons between transcripts (Bowen, 2006; Strauss and Corbin, 2015). To “create order” (Spencer et al., 2003) within the large dataset, transcripts were thoroughly coded and categorized into themes. Initial transcripts were uploaded to NVivo 12 by the second author and charted on a framework matrix, allowing authors to assess the distribution of data across the sample, compare responses, and identify gaps in the data which could be captured in further interviews. As further interviews took place, new codes were added, and existing codes amended. In this way, development of theory paralleled the changes in stakeholders’ experiences of and responses to the pandemic.

The different terminology used by organizations for the Link Worker/Social Prescribing Advisor role should be noted. Going forward, we will refer to advisors attached to GP surgeries as Link Workers, those associated with third sector organizations (who also received referrals from doctors) as Social Prescribing Advisors and those working in rural and remote Scotland as Community Navigators or CNs. When discussing the role in general or across the three schemes, we will continue to refer to them as Link Workers/Social Prescribing Advisors.

### Ethics

All parts of the study were approved by the University of Westminster Research Ethics Committee. All participants were supplied with participant information sheets and gave their consent to interviews being recorded and for extracts of interview data to be used. In all cases, the nature of the study was verbally summarized to participants, along with the content of the consent form, before interviews proceeded. Participants were also reminded that they could withdraw from the interview at any point without any consequences. Our study did not involve issues relating to personal and/or sensitive data. All data use adheres strictly to the terms of the 2018 Data Protection Act. The specificity of some stakeholder roles could allow for easier identification. To mitigate against this, we have elected to publish just short extracts from the interviews, and to use generic identifiers rather than names. Where identification remained possible (as with managers and researchers), the potential for identification was discussed in advance and there were no objections raised.

## Results

Our findings are presented under the following broad headings: demographic and geographical challenges and opportunities; the social prescription; resourcing issues and other uncertainties. We have chosen these headings for narrative purposes and to highlight the responses and issues raised by different stakeholders in our study as they relate to social prescribing and green social prescribing during COVID-19 and its aftermath.

### Demographic and Geographical Challenges

Social prescribing schemes operate in many different physical and social locations, each presenting its own challenges and opportunities. Neither Scotland or England are listed on the Global Multidimensional Poverty Index (United Nations Development Programme, 2020), however, areas of relative poverty and deprivation exist in both countries. A number of the Link Workers and GPs we interviewed in Scotland worked in “Deep End” primary care practices, so named due to their location within the 10% most socioeconomically deprived data zones in Scotland (Mercer et al., 2019) and ranking high on the Scottish Index of Multiple Deprivation (Scottish Government, 2022). Similarly, the GP and Link workers in NE England worked in neighborhoods which fell within the top 10% of the English Indices of Deprivation (Scottish Government, 2022).

One Deep End GP explained how a group of GPs and academics had come together over a decade earlier to “marry academia with what’s going on at the frontline, to get a better perspective on health equalities that were often seen as an abstract thing.” *The Deep End Links Worker Programme* had first been tested in Glasgow GP surgeries, but has since been adopted in other areas. Social prescribing was regarded as integral to the Deep End goal of mitigating health inequalities, but much work was required before any such aim could be realized. Deep End GPs and Link Worker practice lists included generations of families who were multiply disadvantaged, as this Link Worker explained:

We are working with loads of people that have maybe got substance abuse problems, because we are based in the communities where that is more prevalent that will be more prominent…And also ethnic minorities and marginalized groups. There’s a growing Roma community up in [area name removed]- we’re based in three practices there and they’re a very marginalized group. *Link Worker, Glasgow*

Glasgow City Council has outlined a range of sweeping regeneration plans aims at transforming communities (Glasgow City Council, 2022) and stakeholders operating in urban areas emphasized the importance of utilizing existing local green spaces for community wellbeing. One GP who worked closely with local schools quoted statistics suggesting that (although changes in demographics played a part), the green ecology of the area may have contributed to less than severe levels of conduct disorders in school entry children:

It’s interesting because [this area in Glasgow] has got a lot of green space because it’s an older area…they didn’t just shove up lots of housing…There’s a real architectural infrastructure and ecological structure to it as it were, you’ve got a park space, so… what they found was the rates [of conduct disorders] in deprived areas were about double the rates in well-off areas- but it was still a relatively reasonable number. *GP, Deep End Practice, Glasgow*

There was also optimism about the growth of green exercise schemes such parkrun. One parkrun volunteer eulogized about the ways in which parkrun had positively impacted on her local community. The popularity of this particular parkrun was a source of local pride and, by attracting national and international participants into the area, was helping to regenerate a struggling community.

At the same time, stakeholders in urban Scotland recognized that tackling health inequalities was a huge task. The Scottish Index of Multiple Deprivation 2020 (SIMD) showed Glasgow to be the most deprived city and local authority area in Scotland (Scottish Government, 2022). While remaining committed to the ethos of social prescribing, one researcher familiar with the Deep End scheme, had concluded that by itself, social prescribing would never be enough to reverse the Inverse Care Law, whereby those with greatest health and social need live in the areas with lowest levels of care (Marmot, 2018). What was needed, this researcher said, was “the regeneration of areas and you need the other support systems in place.”

An ecological benefit for social prescribing stakeholders in North East England was the location of schemes close to several lakes and an extensive coastline. Speaking about the great local resources in her area, one Link Worker explained how, in her previous role as a health and wellbeing officer, she would regularly accompany individuals and groups of young people to nearby open waters; “we did a lot of boating and kayaking and we…took some school sessions.” Since being part of a GP Link Worker scheme, most of the referrals were for clients over 60, and some were unable to travel to the nearby coast due to limited transport systems:

We live along the coast and I did have one person say, ‘when I’m well enough I want to go along the beach, but I couldn’t get there’ and I said ‘there must be a bus’… And when I checked there isn’t, so transport problems… if you had any mobility problems whatsoever you can’t get there, that worried me a little bit. *Link Worker, NE England*

Rural and remote Scotland is famous for its beauty and open spaces, attracting numerous visitors to its mountains, moors, and beaches. For residents and those working in the area, its physical features can also pose considerable challenges. Alongside an aging society, depopulation has led to an increase in those living alone and with chronic health conditions, and unpaid family care had also declined (Western Isles Integration Joint Board, 2021). Recent census data suggests that the Western Isles has the greatest proportion of lone pensioner households in Scotland (Official National Statistics, 2018) which, even without social isolation or shielding, presents a significant care-package demand. Another complication identified by a CN manager was recruiting staff who were familiar with the geography and amenities in remote communities. CNs operated in several distinct areas/islands, “each with similar problems but unique challenges and differing organizations and infrastructure in place,” thus the job required good local knowledge. According to this CN, one round trip could last a day and travel conditions could be tough:

I did cancel visits the day that we had quite a bit of snow because of just the safety aspects. The roads here, some of them are so tight and windy and they don’t always get gritted if the conditions are going to be poor. *CN, Western Isles Scotland*

### The Social Prescription

Stakeholders described a wide range of activities and interventions which could come under the umbrella of social prescribing. The social prescribing approach was highly individual, as this Link Worker explained; “We work in a completely person-centered way, we don’t have anything formal. We have a kind of personal plan but it’s very conversational.” While GPs could also “do” social prescribing, Link Workers were valued for their flexible approach and broad knowledge of non-medical services available in the local area. One GP in NE England explained how their Link Worker had built bridges between practice staff and third sector organizations:

As a practitioner it’s very difficult to know what organizations do, what sort of a reception people get when they go and what’s out there, and having the Link Worker has really facilitated us…once a week we have different community organizations coming into the health center…and patients can come by and look, but also GPs and the practice [staff]go out. *GP, NE England*

Link Workers attached to health practices considered that their close connection to GPs allowed them to build “good strong relationships with GPs, the practice nurse [etc.]” and (outside of COVID-19) to attend practice meetings. A kind of virtuous circle was described, whereby the presence of Link Workers reminded GPs and practice staff about social prescribing and Link Workers felt acknowledged and valued. On the other hand, Social Prescribing Advisors working outside of the NHS framework emphasized their location close to community amenities which, from the client’s perspective, placed social prescribing “outside of the medical system.”

Previously, we have pointed out the importance of “GP buy-in” to the success of social prescribing (Fixsen et al., 2020), and the degree of GP interest in nature-based pursuits came through came through as a strong factor in its local promotion in this study. Thus, while all the GPs we interviewed spoke favorably about Link Workers/Social Prescribing Advisors, those who were “green participants” themselves were far keener to recommend a specific activity to their patients. One GP we interviewed had been involved in parkrun for decades and routinely recommended it to both patients and staff, as well as giving talks to GP organizations about its benefits. It was his firm opinion that literally everyone would benefit from joining in a local parkrun in different capacities:

You’ve got the obvious benefit of physical activity and for mental health that’s a kind of no brainer… parkrun is brilliant at reaching out to people on the peripheries of our communities, gathering them in with open welcome arms and making them feel included. *GP, NE England*

Another GP/parkrun director talked about the potential benefits of regular outdoor exercise for chronic pain patients and thus for reducing dependence on prescription drugs such as strong painkillers. Nevertheless, working in an area of high social and economic deprivation in Scotland, this GP recognized the complex social and domestic problems many of her patients faced. Patients’ notions about the role of the GP role as there to prescribe medications were deeply entrenched in western society, hence being offered advice on physical exercise as a drug-free alternative was not always greeted with enthusiasm.

During data collection for our study, much of GP time had been taken up with COVID-19 related matters and GPs acknowledged spending limited time attending to the psycho-social needs of their patients. Becoming involved in anything outside of primary care could also seem like too much of a commitment. A parkrun director who had been contacting local GP centers about linking up with a newly opened parkrun noted some reluctance from GPs to get involved due to concerns about taking on extra work; “She [the GP] was hesitant because she thought that it might be like a big commitment for her which is the opposite of what it’s supposed to be.”

For those not ready or able to take up running, walking groups were considered to be a good option, and had remained feasible while indoor activities were closed. Building on this idea, some Link Workers and Social Prescribing Advisors had been offering their clients Walk and Talk sessions in a park or other green space:

Obviously, our hand was forced around the walking and talking but…I had some really lovely sessions with people where we would go… We’ve got some lovely parks in [their local area], the park in my area is lovely and green, so you would do walks around. *Link Worker, NE England*

During COVID-19, social prescribers themselves had to endure greater social and work isolation, and some described working from home in less than ideal conditions. One Link Worker spoke of the surprisingly positive effect of Walk and Talk sessions on her own mental and physical wellbeing:

Honestly the impact on my life doing this job it’s funny because when you’re talking to people about being healthy and well and being mindful you kind of have to reflect as a practitioner and go – and I’m now actually doing these things myself. *Link Worker, NE England*

A further option for encouraging people to spend time out of doors in a social capacity is volunteering. Participants with links to parkrun spoke of the huge benefits of volunteering, in terms of social interaction and sense of purpose. Other outdoor volunteering activities mentioned included local gardening schemes and helping out with park maintenance. This parkrun director cited the example of an elderly man who had been recently bereaved and had been socially isolated by the pandemic, but who had become a regular volunteer at the local parkrun since it re-opened after lockdown:

…and [for this man] volunteering for parkrun has literally been a life saver. When I walk him home after parkun he’s energized, full of beans, full of happy hormones, chatting about who he has met, it gives him a purpose. *parkrun director, NE England*

### Resourcing Issues and Other Uncertainties

While Link Workers generally advocated green social prescribing, they were circumspect about when and to whom they suggested outdoor activities. The Link Worker was often a client’s first point of call, so building a trusting relationship was paramount. The pandemic led to an increase in domestic violence and mental health crises, which had been a major focus for some Link Workers in the Glasgow area:

The referrals coming through are…more round mental health -I know that was prominent right at start but it’s kind around social isolation; the groups are not face-to-face, and a lot of people are digitally excluded for one reason or another. So a lot of high distress calls- people fleeing violence, relationships breaking down. *Link Worker, Glasgow*

A further problem identified concerned the psychological effects of the prolonged period of physical and social isolation attributable to the pandemic. One Link Worker explained how a lot of her clients found even getting out of their homes very difficult; “so the thought of doing a run even though it’s a very accessible park it’s just daunting.” Another Link Worker was of the opinion that pandemic conditions had greatly exacerbated client anxieties and issues:

It doesn’t have to be a real agoraphobia I’m just thinking about anxiety at the thought of going out and COVID has obviously enhanced that… it has just literally terrified people… and people don’t even know what it is they’re interested in anymore they can’t comprehend what the world could be like outside of the home. *Link Worker, NE England*

The client-base of CNs in rural and remote Scotland were predominantly over 65 with one or more long term health condition. One CN listed the principle reasons for referrals as: “Boredom, isolation, anxiety, a lack of technical savvy, that sort of thing.” Part of her work was to link clients to an exercise class or “get them out of the house, even out shopping.” But local travel had been subject to cuts even before COVID-19: “A lot of the people that I spoke to were missing (the bus service) terribly, even before lockdown.” Link Workers in urban areas tended to see people of a more mixed age range, nevertheless they too had clients who were physically frail. As this Link Worker phrased it: “Sometimes the mind is willing, but the body isn’t -you know- so that can sometimes be tricky.”

Although having grown rather accustomed to the “new normal,” the first year of COVID-19 in Deep End GP practices had been “very, very intense.” GPs had been finding remote working difficult, and it had taken a while for Link Workers to get digitally connected to primary care systems. Schemes had been occupied “with frontline work” such as delivering food and medicines and offering one-to-one session with clients, but with many community services in Scotland and England closed temporarily or permanently, referrals to third sector agencies had been limited. For example, Link Workers in Glasgow noted the closure of a domestic abuse service and youth program, while CNs in rural and remote Scotland mentioned the closure of a popular luncheon club. The upheavals and health concerns generated by COVID-19 had understandably reduced the number of volunteers who were coming forward. As this Link Worker explained: “The level of support that we’re giving [since COVID-19] is different because a lot of community organizations in Glasgow have just disappeared.” The situation in the North of England was much the same; “When the services are so lacking it just makes it extra hard.”

Concerns were tempered by the fact that programs such as parkrun had now re-opened and local communities were coming together to fill any voids and meet their local needs. The establishment of new branches of the *Men’s Sheds Association*^2^ following COVID-19, which offered a mixture of indoor and out of door activities and should, according to one Link Worker, encourage more men into social prescribing and rebalance their predominantly female client base. Another Link Worker spoke enthusiastically about a “Couch to 5 km” scheme which a colleague of theirs had recently received funding for in their local area. Despite these new ventures, stakeholders interviewed at the end of 2021 saw a way to go before some people could feel truly comfortable and safe to socialize in large groups, even in open air spaces.

## Discussion

Our study set out to explore the perspectives of different stakeholders involved in the delivery of social prescribing – and notably green social prescribing – during the COVID-19 pandemic. Our stakeholders were in general agreement about the benefits of social prescribing, including nature-based interventions. The latter had been assisted by urban regeneration and popular schemes such as parkrun, but hindered by embedded social and health problems. The scheme serving older people with chronic health conditions in remote areas largely emphasized digital and community connections. Conditions had changed with the pandemic, and stakeholders’ aspirations had needed to be balanced by a pragmatic approach to supporting clients and health services. Near the start of the pandemic, struggling health and social services had led to Link Workers assuming front line roles, such as delivering medicines and food deliveries, while social distancing had meant that Link Workers had been meeting with clients and other staff solely online.

By the close of our study, stakeholders were seeing an easing of restrictions and the number of referrals was increasing, however, the Link Workers we spoke with were faced with a reduced number of local projects to which they could refer their clients. On-going concerns included identifying and supporting disadvantaged and vulnerable groups and the reduced capacity of statutory and third sector services. The rest of the discussion will be concerned with following issues; the redefining of public space since COVID-19 and the capacity of green prescribing to tackle mental health and reduce health inequalities.

### Redefining Public Space Since COVID-19

A key feature of COVID-19 – with long term implications – has been alterations in legal, and personal views and perceptions of public and social space. The NHS webpage on green social prescribing stresses the importance of being outdoors for people’s mental and physical health, and for equality of access to green spaces (NHS England, 2022b). Yet, the creation of healthy environments is complex, with population health closely related to socioeconomic conditions (Costa et al., 2021). Also, for much of the past 2 years, people have been urged to stay indoors or stay local as much as possible. En masse accessing of natural beauty spots including lakes and beaches has provoked alarm (The Guardian, 2020), while recent flouting of lockdown rules in a politician’s private garden has been met with public outrage (Mason et al., 2022). Whilst COVID-19 restrictions have relaxed, our study supports mounting evidence concerning the detrimental mental health effects of the pandemic on many people including the elderly (Fiorillo and Gorwood, 2020; Philip and Cherian, 2020), which may make leaving home and socializing with others a difficult undertaking for months or years to come. Those who have been most strongly advised to stay at home, the chronically ill and/or elderly, are two categories of people for whom social prescribing is frequently recommended (Moffatt et al., 2017).

A further issue concerns the municipal provision of “COVID-safe” public spaces. The availability of green space varies considerably between different urban areas, with no universal standards existing to detail the optimal amount or characteristics of such green space (Lee and Maheswaran, 2011). In one study of green social prescribing, GPs cited proximity to green space, along with availability of nature-based organizations (NBOs) as key motivators in referring patients to green social prescribing (Robinson et al., 2020). Yet, the cost implications of extending green space in urban areas are considerable, especially as concepts of “open space” themselves will now need to be reconsidered with pandemic conditions in mind. This would need to be borne in mind by those promoting green social prescribing in less affluent countries, and in particular those with multidimensional and monetary poverty (United Nations Development Programme, 2020). Empirical evidence on the outdoor spread of COVID-19 is still being gathered, however, it seems likely that facilitation of effective physical distancing, alongside policies encouraging green exercise, will require towns and cities in the future to allocate more surface area to open public spaces (Sharifi and Khavarian-Garmsir, 2020). All these socioecological factors needs to be factored in by governments when promoting green space activities.

### Green Prescribing and Mental Health

A key goal of green social prescribing set by out by NHS England is that of tackling the negative mental health impact of COVID-19 (NHS England, 2022b). Evidence from one meta study found that mentally ill people experienced greater changes in self-esteem following green exercise than other adult population groups (Barton and Pretty, 2010). Good access to green recreational spaces appears to be “equigenic,” meaning that it is able to disrupt the usual conversion of socio-economic inequality related to mental health inequality (Mitchell et al., 2014). Green space has been associated with stress reduction in deprived areas (Ward et al., 2012), while a recent DEFRA study highlights the human health and well-being benefits of exposure to coastal environments including lower levels of mental distress (Department for Environment, Food and Rural Affairs [DEFRA], 2019). Social prescribing is in line with United Kingdom government policy of extending services beyond treating people only when they are ill to keeping them healthy and independent (Friedli et al., 2008). The existence of green prescribing schemes could reduce pressure on GPs who face significant changes in treatment options for mental health conditions such as depression and anxiety as well as social isolation problems (Elsey et al., 2016). In this sense, our findings align with literature suggesting that navigating people toward spending time with others in open green spaces has the potential to alleviate some of the negative mental health effects compounded by the pandemic, such as loneliness, stress, and depression (Aerts et al., 2018; Robinson et al., 2020; NHS England, 2022b).

At the same time, the health impact of COVID-19 across communities and age groups has been very different, and for urban dwellers the impact been particularly uneven. One study found the high incidence and severity of COVID-19 in minority groups to be associated with multiple socioeconomic, cultural and genetic factors, carrying on negative health trends that pre-existed (Khunti et al., 2020). Other serious issues impacting on mental health and wellbeing during COVID-19 are unemployment and financial concerns, domestic abuse, and violence (Office for National Statistics, 2020; Sharifi and Khavarian-Garmsir, 2020). All these problems had been reported by clients of stakeholders in our study and are likely to act as deterrents to taking up green social prescribing.

The heightened health and social anxiety of some clients reported by Link Workers is entirely reasonable, given how those who are older and/or have a disability have been most severely hit by the virus. Living alone, as under social-distancing measures related to COVID-19 can further decrease health and wellbeing (Kamin et al., 2021), and increase the risk of dementia, depression, and premature mortality (Baker and Irving, 2016). As for people living in remote, rural areas, living surrounded by nature has been particularly idealized during the pandemic, however, studies suggest that those living on rural communities are especially likely to experience social isolation compared to urban dwellers (Monteith et al., 2021; Western Isles Integration Joint Board, 2021). While time may change these trends to some extent, it seems important that both GPs and Link Workers receive sufficient training in understanding the psychological and social problems of particular communities, including how to detect and address social anxiety and agoraphobia-like mental health conditions exacerbated by COVID-19. In addition, we recommend that schemes increase their efforts to make green social prescribing more attractive, accessible, and relevant to people from Black, Asian, and Ethnic Minority backgrounds.

### Green Prescribing and Health Inequalities

Another goal set out by NHS England for green social prescribing is that of reducing health inequalities (NHS England, 2022) which, despite various governments pledging to narrow the gaps, continue to be a feature of United Kingdom society. Reducing health inequalities has long been an aim of social prescribing, with schemes such as the Deep End Links Worker Programme purposely located in areas of high social deprivation or disadvantage (Mercer et al., 2019; Hassan et al., 2020). There is optimism that, by with referring people to a broad range of support services, social prescribing can play a key role in helping people to tackle social determinants bound up with health inequalities (Kings Fund, 2018; Islam, 2020).

A counterview is that the concept that social prescribing can reduce health inequalities fails to stand up to critical scrutiny (Bickerdike et al., 2017; Mackenzie et al., 2020; Chng et al., 2021; Gibson et al., 2021). Critics argue that any intervention which persists in individualizing social problems and advocates individual behavior is operating in the realm of fantasy (Mackenzie et al., 2020), and that social prescribing is more likely to result in increased gaps in health and social outcomes (Gibson et al., 2021). A consistent finding of health geography studies is that those residing in socioeconomically deprived neighborhoods have worse health outcomes in comparison to their counterparts (Chaparro et al., 2018). Qualitative research on social prescribing service users in areas of mixed socioeconomic status found those with more income already in possession of good social capital and far less likely to encounter the kinds of major obstacles or set-backs of less privileged service users (Fixsen et al., 2020, 2021; Gibson et al., 2021). With those in the most socially economically deprived areas still receiving the lowest quality of healthcare (Marmot, 2018) and less able to access green amenities, only widespread policy changes can really make a difference to inequitable health provision and outcomes, and this would take decades to achieve.

With reference to green prescribing, the argument that nature is freely available to everybody holds some value, however, relationships between access to green space and its actual use are complex and modified by social deprivation quality, size, and proximity of available green space (Robinson et al., 2020). A variety of factors need to be considered, such as: do people have a garden they can easily and safely access, how far do they live from a park, how easy is it for them to access public transport? The United Kingdom is one of the most car dependent countries in Europe, but only 78% of households own a car. Those in the poorest areas not only suffer from the lowest quality of medical care, they also suffer the most in terms of air pollution and lack of easy access to green spaces (Greenpeace, 2021). Intersecting inequalities are shaped by social challenges and disruptions often for the worse rather than the better (Ward et al., 2012; Fixsen et al., 2021). COVID-19 has exposed inequalities and social fault lines already in existence in society, with people at the bottom of the socioeconomic spectrum hit disproportionately (Sharifi and Khavarian-Garmsir, 2020).

A further issue concerns stigma associated with low socioeconomic status and health problems, such as obesity. External and internal forms of stigma (Goffman, 1963) linked with mental health and visible disabilities have been widely studied and documented (Corrigan and Rao, 2012). When accessing outdoor spaces, people are, and may see themselves, as at higher risk of social judgent and abuse. Social divisions and stereotypes can act as barriers to participating in outdoor activities like running, due to its association with certain body ideals, the requirement of particular clothing and potential derogation of slower participants (Hindley, 2020). The inclusive and anti-discriminatory ethos of parkrun can help to reconcile the paradox of being an “unfit runner” (Wiltshire et al., 2018, p. 3). Nevertheless these positive messages have to contend with deep seated attitudes and prejudices concerning behaviors and body shapes (Sikorski et al., 2011; Corrigan and Rao, 2012), while challenges remain in terms of increasing the cultural and ethnic diversity of interest in and membership to outdoor activity groups (Fullagar, 2016).

None of this is to detract from the work done by the stakeholders featured in our study, nor the many local social prescribing services which set out to deliberately target those with highest socio-economic need and to attract an ethnically and socially diverse clientele. Rather, we make these points as a reality check and to point out the Herculean task those delivering green social prescribing face in attempting to lessen structurally embedded health inequalities.

## Conclusion

There is much that can be learned from engaging with stakeholders who are involved in delivering green social prescribing in varied locations across the globe. In this study we have focused on stakeholder perspectives of the challenges and approaches to social prescribing in the United Kingdom during the COVID-19 pandemic, to give voice to these stakeholders and to draw attention to the socioecological factors that need to be factored into government and organizational design and promotion of nature-based activities. Limitations to our study include sample size and other factors mediated by limited funds and time. Other limitations are those associated with on-line (remote) interviewing and the closure of many third sector services at this time. Originally our study was to include face-to-face focus group interviews with clients of social prescribing, however, travel opportunities and lack of access to NHS patient records during COVID-19 made this impossible within the study frame. Instead, we chose to expand the study to rural Scotland and NE England and include a larger cohort of professional stakeholders in different settings. We also recognize that our recommendations and conclusions relate to countries with sufficient resources and income to offer financial support to social prescribing and community services.

For further study, we would recommend more comparative studies of social prescribing in different socioeconomic localities, and more exploration of green social prescribing in rural areas, where nature-based pursuits might seem freely available, but where the barriers of access may exist in the form of mobility issues, lack of transport, hazardous pathways et cetera. We conclude by stating that green social prescribing needs to be seen as part of a package of national or international social and economic policies to reduce structurally embedded inequalities at every level. The alternative is for green social prescribing to be used as panacea for solving problems and social issues such as loneliness, poverty, and other aspects of inequalities (Drinkwater et al., 2019), which instead require complex and lasting interventions.

## Data Availability Statement

The datasets presented in this article are not readily available because the interviews contain confidential information. Requests to access the datasets should be directed to AF, A.Fixsen@westminster.ac.uk.

## Ethics Statement

The studies involving human participants were reviewed and approved by the University of Westminster Research Ethics Committee. The patients/participants provided their written informed consent to participate in this study. Written informed consent was obtained from the individual(s) for the publication of any potentially identifiable images or data included in this article.

## Author Contributions

Both authors listed have made a substantial, direct, and intellectual contribution to the work, and approved it for publication.

## Conflict of Interest

The authors declare that the research was conducted in the absence of any commercial or financial relationships that could be construed as a potential conflict of interest.

## Publisher’s Note

All claims expressed in this article are solely those of the authors and do not necessarily represent those of their affiliated organizations, or those of the publisher, the editors and the reviewers. Any product that may be evaluated in this article, or claim that may be made by its manufacturer, is not guaranteed or endorsed by the publisher.

## Footnotes

1
https://www.parkrun.org.uk

2
https://menssheds.org.uk
