# Supplementary material for: Challenges and Approaches to Green Social Prescribing During and in the Aftermath of COVID-19: A Qualitative Study
Source: Front Psychol. 2022 May 16;13:861107. doi: 10.3389/fpsyg.2022.861107 (PMC9149572; doi:10.3389/fpsyg.2022.861107)
Supplement: Supplementary file 1 [file Table_1.DOCX]

**Indicative questions for interviews with professionals and volunteers**

1. Can you tell me about your role with the social Prescribing/ community service?
2. Could you describe how the service operates from your perspective?

- Referral system and referral criteria
- Support offered to service users
- How service users ‘exit’ the service

1. In what ways has communication and partnerships between the social prescribing service and the voluntary and community sector been established?
2. What do you see are the key strengths of the social prescribing/ community service model?
3. What are your experiences of and involvement in nature based social prescribing? (cite relevant example here)
4. How has the service adapted to the conditions of the pandemic?
5. How the pandemic conditions impacted on your role in the service?
6. What have been the main challenges in delivering the service during Covid-19?
7. What opportunities has it presented in terms of new initiatives?
8. From your experience, in what ways at all do existing social prescribing services support vulnerable and marginalised members of communities in your area?
9. To what extent has the voluntary and community sector succeeded in providing support for marginalised members of communities in your area, including through nature-based activities?
10. Are there any recommendations you would offer to improve access to nature based social prescribing for vulnerable and marginalised members of communities?
11. How do you see the future of nature based social prescribing?
