# Supplementary material for: Challenges and Approaches to Green Social Prescribing During and in the Aftermath of COVID-19: A Qualitative Study
Source: Front Psychol. 2022 May 16;13:861107. doi: 10.3389/fpsyg.2022.861107 (PMC9149572; doi:10.3389/fpsyg.2022.861107)
Supplement: Supplementary file 2 [file Presentation_1.PPTX]

## Slide 1
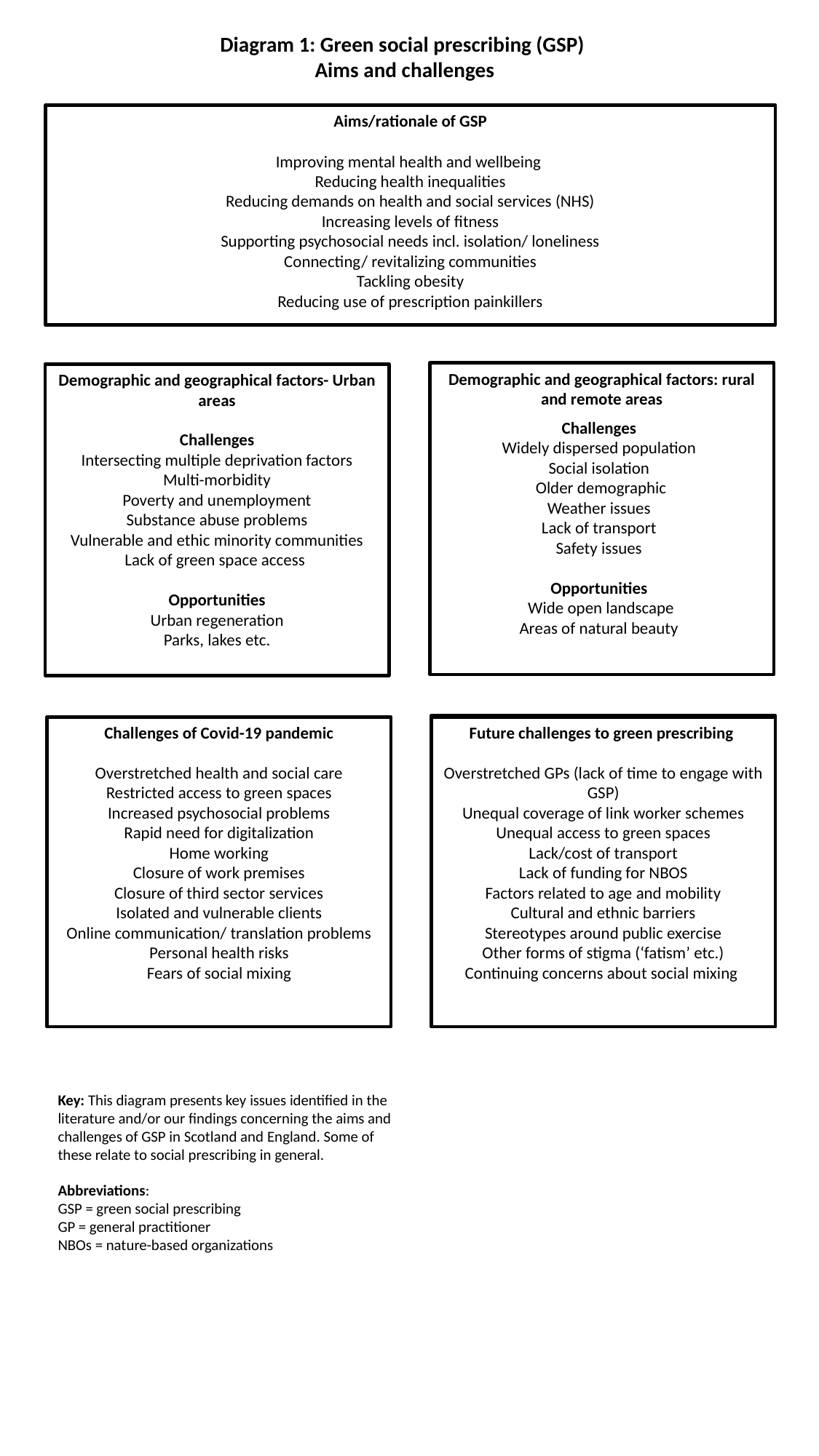

Diagram 1: Green social prescribing (GSP)
Aims and challenges
Aims/rationale of GSP
Improving mental health and wellbeing
Reducing health inequalities
Reducing demands on health and social services (NHS)
Increasing levels of fitness
Supporting psychosocial needs incl. isolation/ loneliness
Connecting/ revitalizing communities
Tackling obesity
Reducing use of prescription painkillers
Demographic and geographical factors: rural and remote areas
Demographic and geographical factors- Urban areas
Challenges
Intersecting multiple deprivation factors
Multi-morbidity
Poverty and unemployment
Substance abuse problems
Vulnerable and ethic minority communities
Lack of green space access
Opportunities
Urban regeneration
Parks, lakes etc.
Future challenges to green prescribing
Overstretched GPs (lack of time to engage with GSP)
Unequal coverage of link worker schemes
Unequal access to green spaces
Lack/cost of transport
Lack of funding for NBOS
Factors related to age and mobility
Cultural and ethnic barriers
Stereotypes around public exercise
Other forms of stigma (‘fatism’ etc.)
Continuing concerns about social mixing
Future challenges to green prescribing
Overstretched GPs (lack of time to engage with GSP)
Unequal coverage of link worker schemes
Unequal access to green spaces
Lack/cost of transport
Lack of funding for NBOS
Factors related to age and mobility
Cultural and ethnic barriers
Stereotypes around public exercise
Other forms of stigma (‘fatism’ etc.)
Continuing concerns about social mixing
Challenges of Covid-19 pandemic
Overstretched health and social care
Restricted access to green spaces
Increased psychosocial problems
Rapid need for digitalization
Home working
Closure of work premises
Closure of third sector services
Isolated and vulnerable clients
Online communication/ translation problems
Personal health risks
Fears of social mixing
Key: This diagram presents key issues identified in the literature and/or our findings concerning the aims and challenges of GSP in Scotland and England. Some of these relate to social prescribing in general.
Abbreviations:
GSP = green social prescribing
GP = general practitioner
NBOs = nature-based organizations
Challenges
Widely dispersed population
Social isolation
 Older demographic
Weather issues
Lack of transport
Safety issues
Opportunities
 Wide open landscape
Areas of natural beauty
